# Primary care utilisation, adherence to guideline-based pharmacotherapy and continuity of care in primary care patients with chronic diseases and multimorbidity – a cross-sectional study

**DOI:** 10.1186/s12875-023-02191-6

**Published:** 2023-11-13

**Authors:** Andy Maun, Cecilia Björkelund, Eva Arvidsson

**Affiliations:** 1 Institute of General Practice / Primary Care, Faculty of Medicine and Medical Center, University of Freiburg, Elsässer Str 2m, Freiburg, DE-79110 Germany; 2 Primary Health Care, Department of Public Health and Community Medicine, Sahlgrenska Academy, University of Gothenburg, Box 454, Göteborg, SE-405 30 Sweden; 3 Research and Development Unit for Primary Care, Futurum, Hus B4, Länssjukhuset Ryhov, Jönköping, SE-551 85 Sweden

**Keywords:** Primary care, Continuity of care, Continuity index, Cross-sectional study, Multimorbidity, Guideline adherence

## Abstract

**Background:**

To understand how to improve care for patients with chronic diseases and multimorbidity we wanted to describe the prevalence of different chronic diseases and the pattern of multimorbidity and to analyse the associations between occurrence of diseases and primary care utilization, adherence to guideline-based pharmacotherapy, and continuity of care.

**Methods:**

Retrospective cross-sectional study of routine care data of the general population in region Jönköping in Sweden (345 916 inhabitants using primary care services) covering 4.3 years.

**Participants:**

Patients fulfilling the inclusion criteria of having ≥ 1 of 10 common chronic diseases and ≥ 3 visits to primary care between 2011 and 2015.

**Primary outcome measures:**

In order to determine diseases and multimorbidity, primary care utilisation, adherence to guideline-based pharmacotherapy, frequencies and percentages, interval and ratio scaled variables were described using means, standard deviations, and various percentiles in the population. Two continuity indices were used (MMCI, COC) to describe continuity.

**Results:**

Of the general population, 25 829 patients fulfilled the inclusion criteria (7.5% of the population). Number of diseases increased with increasing age, and multimorbidity was much more common than single diseases (mean 2.0 per patient). There was a slight positive correlation (0.29) between number of diseases and visits, but visits did not increase proportionally to the number of diseases. Patients with physical diseases combined with anxiety and/or depression made more visits than others. The number of diseases per patient was negatively associated with the adherence to pharmacotherapy guidelines. There was no association between continuity and healthcare utilisation or adherence to pharmacotherapy guidelines.

**Conclusions:**

Multimorbid patients are common in primary care and for many chronic diseases it is more common to have other simultaneous diseases than having only one disease. This can make adherence to pharmacotherapy guidelines a questionable measure for aged multimorbid patients. Existing continuity indices also revealed limitations. Holistic and patient-centred measures should be used for quality assessment of care for multimorbid patients in primary care.

**Supplementary Information:**

The online version contains supplementary material available at 10.1186/s12875-023-02191-6.

## Background

The number of persons with multimorbidity (multiple health conditions, often permanent requiring complex and ongoing care) is steadily increasing and has been reported to exceed 65% among those 65 years and older [1–4]. For these patients, who are characterized by a lower health-related quality of life and reduced functional capacity, person-centred care is essential and currently predominantly a task of primary care providers [5–7].

For patients with chronic diseases “drug therapy according to guidelines”, is often used as measures of quality. Examples are the proportion of patients with diabetes treated with statins or patients with atrial fibrillation treated with anticoagulants [8–12]. However, this type of measurements may be relevant for outcomes of single chronic diseases, but their importance for patients with multimorbidity remains unclear, as there is a need to adapt therapies to – and with – the individual and to avoid interactions and polypharmacy [5, 7, 13]. Furthermore, for patients with multimorbidity, frequent hospitalisation, often due to medication related problems, adds to reduced quality of life and increased costs [14].

Much effort is currently being put into improving management for persons with multimorbidity [5], including clinical guidelines, both creating new for patients with multimorbidity and adapting existing ones for single diseases [15, 16]. Effective management and high quality of health services is crucial as several studies indicate a curvilinear, near exponential, relationship between multiple chronic diseases and costs, partially caused by the higher number of care providers involved [17–19].

Continuity has shown a positive correlation with improved preventive care, reduced hospitalisation, significantly lower healthcare utilisation and costs, better treatment effects, more satisfied patients, fewer sick leaves and referrals, and reduced drug consumption [17, 19–23].

*Relational continuity is* the long term relationship between the patient and a health professional, but also *management continuity, (*the consistent and coherent management of a health condition between different phases of the disease and various levels of care) and *informational continuity, (*the persistent access to, and use of, health related information) are important for quality of care [24, 25].

To understand how to improve care for patients with chronic diseases and multimorbidity, we need more accurate ways to describe their heterogeneity and the healthcare utilisation. In this study we epidemiologically describe a defined population in a Swedish region concerning prevalence of different chronic diseases and multimorbidity, primary care utilisation, the adherence to guideline-based pharmacotherapy, continuity of care, and the association between them.

## Methods

### Aim

The aim was to describe the prevalence of different chronic diseases and the pattern of multimorbidity in a population with chronic diseases and contacts with primary care, and to analyse the associations between occurrence of diseases and a) primary care utilization, b) adherence to guideline-based pharmacotherapy and, c) continuity of care.

### Setting

#### Primary care in Sweden

Swedish Family Physicians or General Practitioners (GPs) have undergone 5 years of specialisation after 5½ years of university studies and 18–21 months of internship. About 15–20% of all specialists are GPs. Three consultations with a specialist per inhabitant a year is average [26]; half of these are with a GP. Consultations with GPs are, on average, 20 min.

GPs often work at primary health care centres (PHCC) in close collaboration with practice nurses and other health care personnel; however, there are also a few single doctor practices. There are roughly 1200 PHCCs (or GP practices) in Sweden (population 10.5 million). In Sweden, the specialists in primary care are almost exclusively specialists in Family medicine, i.e. GPs. However, in our calculations we included all doctors at all health centres.

Almost all primary health care is publicly financed through taxes. Around 40% is privately provided and 60% publicly provided. The reimbursement systems differ between the 21 regions in Sweden, but in each region, the reimbursement is by law the same for privately and publicly provided primary health care. The most common reimbursement system is capitation, i.e. that PHCCs are financed in proportion to the number of patients registered at the PHCC. A small share can also be related to number of visits and to achieved quality goals. The capitation is often related to age, Adjusted Clinical Groups (ACG) [27, 28] and Care Need Index (CNI) [29] of the listed patients. The ACG system reflects burden of disease of the registered patients by the combinations of their diagnoses. All inhabitants are free to choose any PHCC, and the patient fees are low and the same for privately and publicly produced primary care. While most PHCCs try to provide a high continuity between patients and specific doctors, not all PHCC are able to achieve this goal.

### Design and material

This is a retrospective cross-sectional study based on data from electronic medical records (EMRs) concerning patients registered with all of the PHCCs in a region Jönköping in southern Sweden from 2011 to 2015. The entire population in the region was 347 837 persons 2015, of which 345 916 persons (99,4%) were registered with a PHCC, which made data on diagnoses, number of visits, which GPs the patient had visited, and prescriptions accessible. All the PHCC used EMRs for registration of patients and patient contacts, morbidity recording, and prescriptions during the study period. The ICD-10 classification system was used for diagnosis registration.

Patients fulfilling the following inclusion criteria were selected of further analysis:

- Diagnosed with one or more of 10 selected chronic diseases in 2011: dementia, depression, anxiety, diabetes, atrial fibrillation, heart failure, ischaemic heart disease, COPD, stroke/TIA and/or vascular diseases other than ischaemic heart disease and stroke/TIA (but included in the definition of CHA_2_DS_2_VASc). (For diagnosis codes, see Additional file 1: Appendix 1).
- Visited a general practitioner (GP) at least 3 times during the study period.
- Still registered with a PHCC in the region at the end of May 2015.

The selection of the diagnoses was made to include the most common chronic diseases that are regularly monitored in Swedish primary care. Patients with these diseases normally visit their GP for at least an annual check-up when e.g., health status is evaluated, medication is adjusted and lifestyle interventions are discussed. The selection of diseases and diagnoses was made in consensus by three of the investigators, all GPs. For each patient only one diagnosis from each disease group was counted, e.g., type 2 diabetes mellitus without complications (E11.9), and type 2 diabetes mellitus with unspecified complications (E11.8) in the same patient was counted as one disease. Multimorbidity has been defined as having two or more diseases at the same time [1], in our study two or more of the 10 selected chronic diseases. Multimorbidity ratio was defined as the number of multimorbid patients with the disease divided by the number of patients with solely the disease. Values < 1.0 indicate a more frequent occurrence of the disease as a single diagnosis while values > 1.0 indicate a more frequent occurrence of the disease as one of several diagnoses. The patients’ diagnoses were registered in EMRs in connection with each visit to the GPs.

Parameters used as proxies for adherence to guideline-based pharmacotherapy was based on Swedish national recommendations on secondary prevention for some chronic diseases during the study period. This included prescriptions of anticoagulants for patients with atrial fibrillation and CHA_2_DS_2_VASc ≥ 2, beta-blockers to patients with ischaemic heart disease and heart failure, statins for patients with diabetes, stroke or TIA, or ischaemic heart disease (for the ATC codes see Additional file 1: Appendix 2).

For calculation of continuity of care, two indices that measure the dispersion of continuity were chosen: MMCI (Modified Modified Continuity Index) and COC (Continuity Of Care) [30]. These indices reflect a managing perspective and quantify the proportion of visits with distinct GPs in relation to all GPs involved. Therefore, these indices focus particularly on the common request that a multimorbid patient should preferably see the same GP most of the visits, which can be described as interpersonal continuity. For both indices values range from 0 (each visit made to a different GP) to 1 (all visits made to a single GP). MMCI also takes into account the number of GPs seen and the number of primary care visits, while COC additionally takes into account the proportion of visits made to each GP. COC is the most used dispersion measure and MMCI is a modification of the first one [30].

At least three visits in the study period (2011–2015) were considered as an indication of need of continuous care and chose continuity indices for three and more visits.

For patients with chronic diseases fulfilling the inclusion criteria, the following data were collected from the EMRs from January 2011 up to May 2015 (totally 52 months): new diagnoses for any of the selected chronic diseases number of visits to a GP, number of different doctors, which GP that was visited each time, prescriptions of anticoagulants, beta-blockers and statins (used as proxies for adherence to guideline-based pharmacotherapy). In addition, CHA_2_DS_2_VASc was calculated for patients with atrial fibrillation [31], (Additional file 1: Appendix 3).

Patient identities were deleted and replaced by code numbers immediately after data extraction. This was performed by a data manager independent from the research group. Consequently, the research group did not have access to patient identities.

### Statistical analysis

Interval and ratio scaled variables were described using means, standard deviations, and various percentiles for different subgroups in the population. Ordinal variables were described using percentiles. Number of patients within different subgroups were described using frequency and percentage. The associations between the number of diseases and the degree of health care utilisation and between degree of continuity of care and the adherence to guideline-base pharmacotherapy was calculated by multiple modelling analysis (Spearman correlation and quantile regression).

The statistical programs used were Stata version 14.3 and Excel version 2011.

### Patient and public involvement

Patients were not involved in development of the research question, design of the study and outcome measures.

## Results

### Prevalence of common chronic diseases and multimorbidity

Of the 345 916 persons registered with one of the PHCCs in the region (99.4% of the total population in the area) 25 829 patients (7.5%) fulfilled the inclusion criteria i.e., had at least three visits to a PHCC during the study period and at least one chronic disease out of 10 different identified diagnosis. The study population represented 0.4% of persons aged 0–29 years, 4.4% of persons aged 30–59 years, 16.6% of persons aged 60–79 years, and 34.8% of persons aged 80 years and older.

Table 1 presents the number of patients with each of the 10 selected diagnoses, including the numbers of patients who solely had the diagnose (A) and those including who concurrently had multimorbidity (B). The number of patients suffering from a certain disease together with at least one more outreached by far the number who had only one chronic disease.

The mean number of diagnoses per patient was 2.0 (median 2). Diabetes was the most common disease, corresponding to a prevalence of 3%, followed by depression (prevalence 2.7%), and ischaemic heart disease (prevalence 2.4%).

**Table 1:** Number of patients with each of the 10 selected diagnoses, and number and percentage of individuals with a single disease, and as one of several diseases, respectively

**Disease (Diagnosis group)**	**Age group**	**Number of patients with the disease**						**Multimorbidity ratio (B/A)**^a^
		**All patients with the disease (= A + B)**		**Patients with solely the disease (= A)**		**Multimorbid patients with the disease (= B)**		
		**Total**	**Percentage of study population**	**Total**	**Percentage of study population**	**Total**	**Percentage of study population**	
		n	%	n	%	n	%	
**Diabetes**	0–29 years	12	< 0.1	8	< 0.1	4	< 0.1	0.5
	30–59 years	1656	6.4	879	3.4	777	3.0	0.9
	60–79 years	6036	23.4	2456	9.5	3580	13.9	1.5
	≥ 80 years	2730	10.6	609	2.4	2121	8.2	3.5
	**total**	**10,434**	**40.4**	**3952**	**15.3**	**6482**	**25.1**	**1.6**
**Depression**	0–29 years	503	1.9	189	0.7	314	1.2	1.7
	30–59 years	3845	14.9	1207	4.7	2638	10.2	2.2
	60–79 years	3188	12.3	612	2.4	2576	10.0	4.2
	≥ 80 years	1880	7.3	136	0.5	1744	6.8	12.8
	**total**	**9416**	**36.5**	**2144**	**8.3**	**7272**	**28.2**	**3.4**
**Ischaemic Heart Disease**	0–29 years	7	< 0.1	5	< 0.1	2	< 0.1	0.4
	30–59 years	493	1.9	162	0.6	331	1.3	2.0
	60–79 years	4288	16.6	1179	4.6	3109	12.0	2.6
	≥ 80 years	3671	14.2	588	2.3	3083	11.9	5.2
	**total**	**8459**	**32.2**	**1934**	**7.5**	**6525**	**25.3**	**3.4**
**Anxiety**	0–29 years	318	1.2	0	0	318	1.2	n.a
	30–59 years	2544	9.8	16	0.1	2528	9.8	158.0
	60–79 years	2211	8.6	46	0.2	2165	8.4	47.1
	≥ 80 years	1308	5.1	4	< 0.1	1304	5.0	326.0
	**total**	**6381**	**24.7**	**66**	**0.3**	**6315**	**24.4**	**95.7**
**Atrial fibrillation**	0–29 years	0	0	0	0	0	0	n.a
	30–59 years	131	0.5	40	0.2	91	0.4	2.3
	60–79 years	1944	7.5	384	1.5	1560	6.0	4.1
	≥ 80 years	2452	9.5	186	0.7	2266	8.8	12.2
	**total**	**4527**	**17.5**	**610**	**2.4**	**3917**	**15.2**	**6.4**
**Heart Failure**	0–29 years	2	< 0.1	1	< 0.1	1	< 0.1	1
	30–59 years	121	0.5	18	0.1	103	0.4	5.7
	60–79 years	1485	5.7	67	0.3	1418	5.5	21.2
	≥ 80 years	2575	10	82	0.3	2493	9.7	30.4
	**total**	**4183**	**16.2**	**168**	**0.7**	**4015**	**15.5**	**23.9**
**Stroke/TIA**	0–29 years	1	< 0.1	1	< 0.1	0	0	n.a
	30–59 years	292	1.1	101	0.4	191	0.7	1.9
	60–79 years	1862	7.2	381	1.5	1481	5.7	3.9
	≥ 80 years	1729	6.7	172	0.7	1557	6.0	9.1
	**total**	**3884**	**15**	**655**	**2.5**	**3229**	**12.5**	**4.9**
**COPD**	0–29 years	1	< 0.1	0	0	1	< 0.1	n.a
	30–59 years	218	0.8	35	0.1	183	0.7	5.2
	60–79 years	1405	5.4	188	0.7	1217	4.7	6.5
	≥ 80 years	598	2.3	40	0.2	558	2.2	14.0
	**total**	**2222**	**8.6**	**263**	**1.0**	**1959**	**7.6**	**7.4**
**Dementia**	0–29 years	0	0	0	0	0	0	n.a
	30–59 years	10	< 0.1	3	< 0.1	7	< 0.1	2.3
	60–79 years	427	1.7	27	0.1	400	1.5	14.8
	≥ 80 years	1343	5.2	113	0.4	1230	4.8	10.9
	**total**	**1780**	**6.9**	**143**	**0.6**	**1637**	**6.3**	**11.4**
**Vascular disease**	0–29 years	2	< 0.1	2	< 0.1	0	0	n.a
	30–59 years	51	0.2	10	< 0.1	41	0.2	4.1
	60–79 years	577	2.2	59	0.2	518	2.0	8.8
	≥ 80 years	571	2.2	40	0.2	531	2.1	13.3
	**total**	**1201**	**4.6**	**111**	**0.4**	**1090**	**4.2**	**9.8**
***Total number of diagnoses***		***52,487***		***10,046***		***42,441***		

*n.a.* Not applicable

^a^ The multimorbidity ratio (B/A) is the number of multimorbid patients with the disease divided by the number of patients with solely the disease. Total number of patients = 25 829

**Fig. 1:**
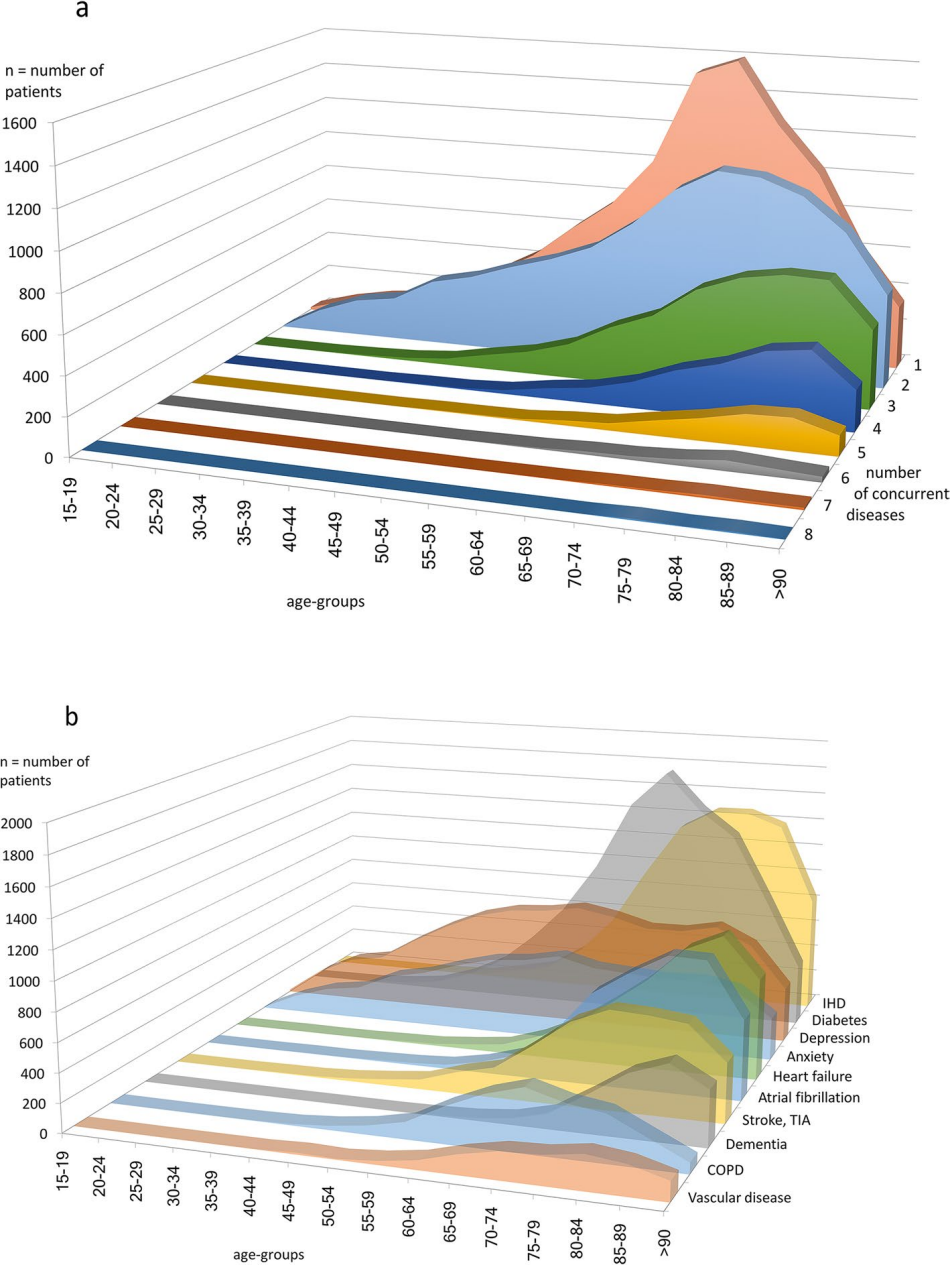
**a**-**b** Number of chronic diseases per patient and multimorbidity in different age groups (**a** number of patients with 1–8 chronic diseases; **b** number of patients with each diagnose for 10 chronic conditions)

Over 60% of the study population (*n* = 25 829) had more than one chronic disease, and the number of diseases per patient increased with increasing age. No patient had more than 8 of the selected diseases (Fig. 1a). Age-specific prevalence varied with depression and anxiety most evenly spread while dementia had its peak among the oldest patients (Fig. 1b).

The most common types of multimorbidity with two diseases, were the combinations of anxiety and depression and the combination of diabetes and ischaemic heart disease - both solely and in combination with other diseases (Table 2). The most common types of multimorbidity with solely three simultaneous diseases were the combination of anxiety, depression and diabetes, and the combination of atrial fibrillation, ischaemic heart disease and heart failure. However, the of number patients who were suffering of further chronic diseases beside these combinations outreached by far the number of patients with solely these combinations of two respectively three diseases.

**Table 2:** Pattern of multimorbidity and prevalence in the study population of individuals with at least 1 chronic disease (*n* = 25,829)

Pattern of multimorbidity (combination of diseases)	Number of patients who had this pattern of multimorbidity and no other chronic diseases	Percentage of study population (%)	Number of patients who had this pattern of multimorbidity *and* further chronic diseases	Percentage of study population (%)
**Multimorbidity with 2 diseases**				
Anxiety and Depression	2 868	11.10	4699	18.19
Diabetes and IHD	985	3.81	2898	11.22
Depression and Diabetes	492	1.90	1931	7.48
IHD and Heart Failure	401	1.55	2438	9.44
Anxiety and Diabetes	395	1.53	1557	6.03
Atrial Fibrillation and Heart Failure	324	1.25	2003	7.75
**Multimorbidity with 3 diseases**				
Anxiety, Depression and Diabetes	391	1.51	782	3.03
Atrial Fibrillation, IHD and Heart Failure	288	1.12	288	1.12
Diabetes, IHD and Heart Failure	225	0.87	910	3.52
Anxiety, Depression and IHD	182	0.70	676	2.62
Anxiety, Depression, COPD	138	0.53	354	1.37
Diabetes, Atrial Fibrillation and Heart Failure	130	0.50	680	2.63

For patients with four diseases the most common types of multimorbidity were diabetes, atrial fibrillation, ischaemic heart disease and heart failure together, as well as anxiety, depression, diabetes and ischaemic heart disease.

**Fig. 2:**
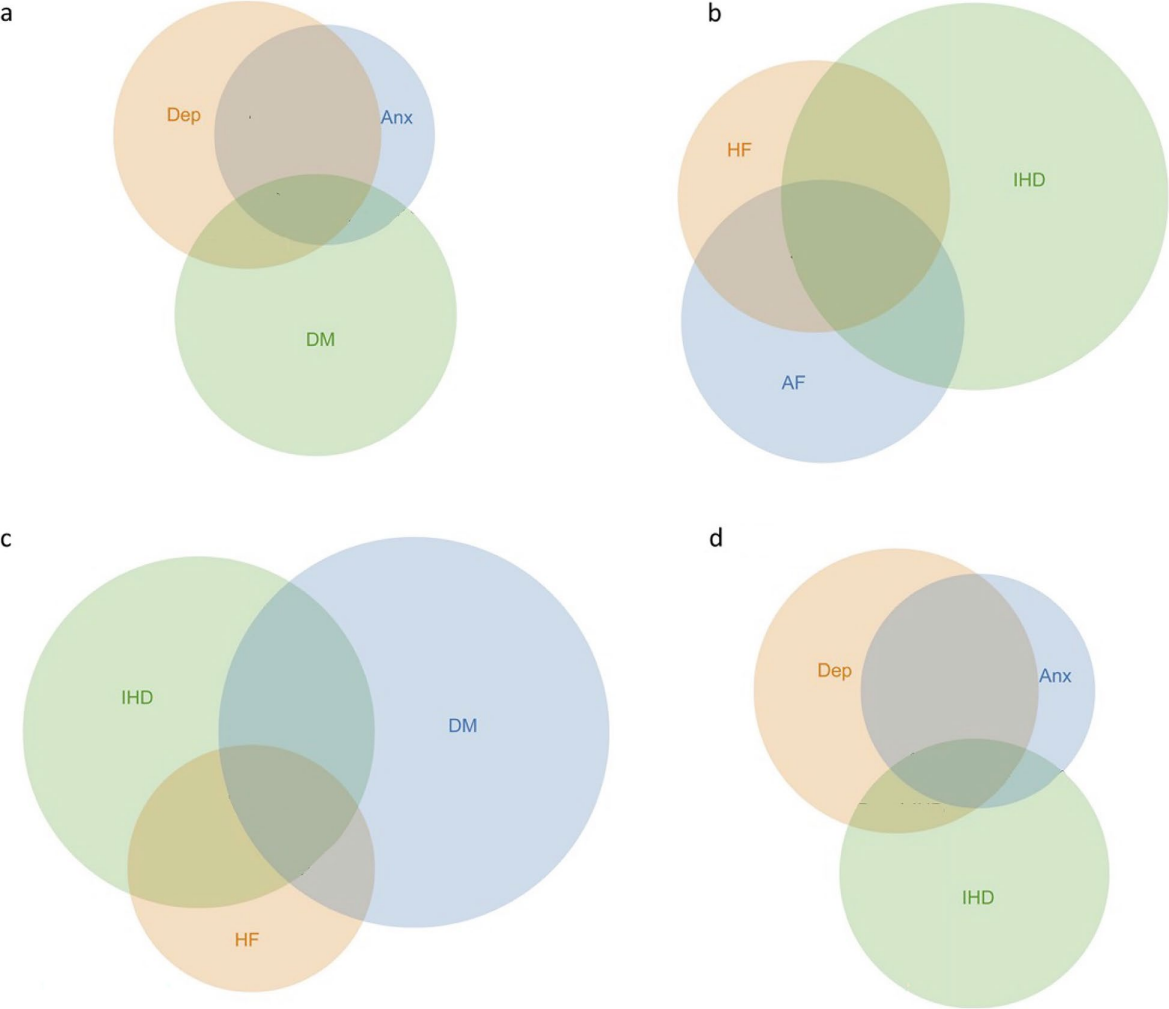
**a**-**d** Multimorbidity for individuals concerning 3 different diseases in the total group of individuals with 1 or more chronic diseases. (**a** Anxiety, Depression and Diabetes, in total 73% of the study population; **b** Atrial Fibrillation, IHD and Heart Failure, in total 46% of the study population; **c** Diabetes, IHD and Heart Failure, in total 66% of the study population; **d** Anxiety, depression and IHD, in total 67% of the study population)

Multimorbidity is also illustrated in Fig. 2a-d, representing the frequent combinations of depression and anxiety, ischemic heart disease and heart failure and ischemic heart disease and diabetes.

### Primary care utilisation

The median number of visits to a GP during the entire study period was 7. However, the range was between 3 and 164, i.e. the majority of the patients made relatively few visits to a GP while a few patients made many visits. Mean number of visits was 9.1 per patient for the entire period, or 2.1 visits per patient per year.

We found a slight positive correlation (0.29; Spearman) between number of visits and number of diseases, but the increase in visits was not proportional to the increase in the number of diseases. For example, the median number of visits for patients with one diagnosis was 7.38 compared to 10.88 visits for a patient with three diseases (Table 3).

**Table 3:** Number of visits for patients with different number of diseases and with common combinations of diseases. Total number of visits, mean number of diseases/year and mean total number of visits 2011–2015 with standard deviations and minimum and maximum values shown

Number of diseases				N	Mean visits/ year	Mean visits total	Median	SD	min	max
1 disease				10,046	1.7	7.38	3	4.57	3	57
2 diseases				9025	2.16	9.34	3	6.09	3	89
3 diseases				3999	2.51	10.88	4	7.31	3	164
4 diseases				1777	2.71	11.74	4	7.42	3	65
5 diseases				705	2.92	12.65	5	7.63	3	72
6 diseases				198	3.34	14.46	4	8.01	3	60
7 diseases				59	3.53	15.32	4	8.83	3	45
8 diseases				20	3.73	16.15	5	7.92	5	30
Anxiety and depression				2868	2.54	10.68	9	7.10	3	68
Diabetes and IHD				985	1.85	7.82	7	4.62	3	43
Depression and diabetes				492	2.31	9.67	8	6.28	3	51
Other with 2 diseases				21,484	2.08	8.97	7	6.01	3	164
Anxiety, depression and diabetes				391	3	12.90	11	7.45	3	43
Atrial Fibrillation, IHD and Heart failure				288	2.54	10.79	9	6.05	3	46
Other with 3 diseases				25,150	2.08	9.05	7	6.14	3	164

A multiple modelling analysis using quantile regression showed that the number of visits varied slightly with different combinations of diseases. Patients with anxiety and/or depression had more visits than patients with combinations of diseases in which anxiety and/or depression were not included.

### Adherence to guideline-based pharmacotherapy

Around two-thirds of the patients with diabetes and/or ischaemic heart disease were prescribed statins during 2014 and/or 2015, while only a little more than half of patients with TIA/stroke had statins. Around 70% of patients with heart failure and/or ischaemic heart disease were prescribed beta-blockers. Out of all patients with atrial fibrillation, almost 88% had CHA_2_DS_2_VASc ≥ 2 (i.e. indication for anticoagulant therapy), and 69% of these were prescribed anticoagulants during 2014 and/or 2015. For patients with CHA_2_DS_2_VASc < 2 the proportion treated was 67%.

**Fig. 3:**
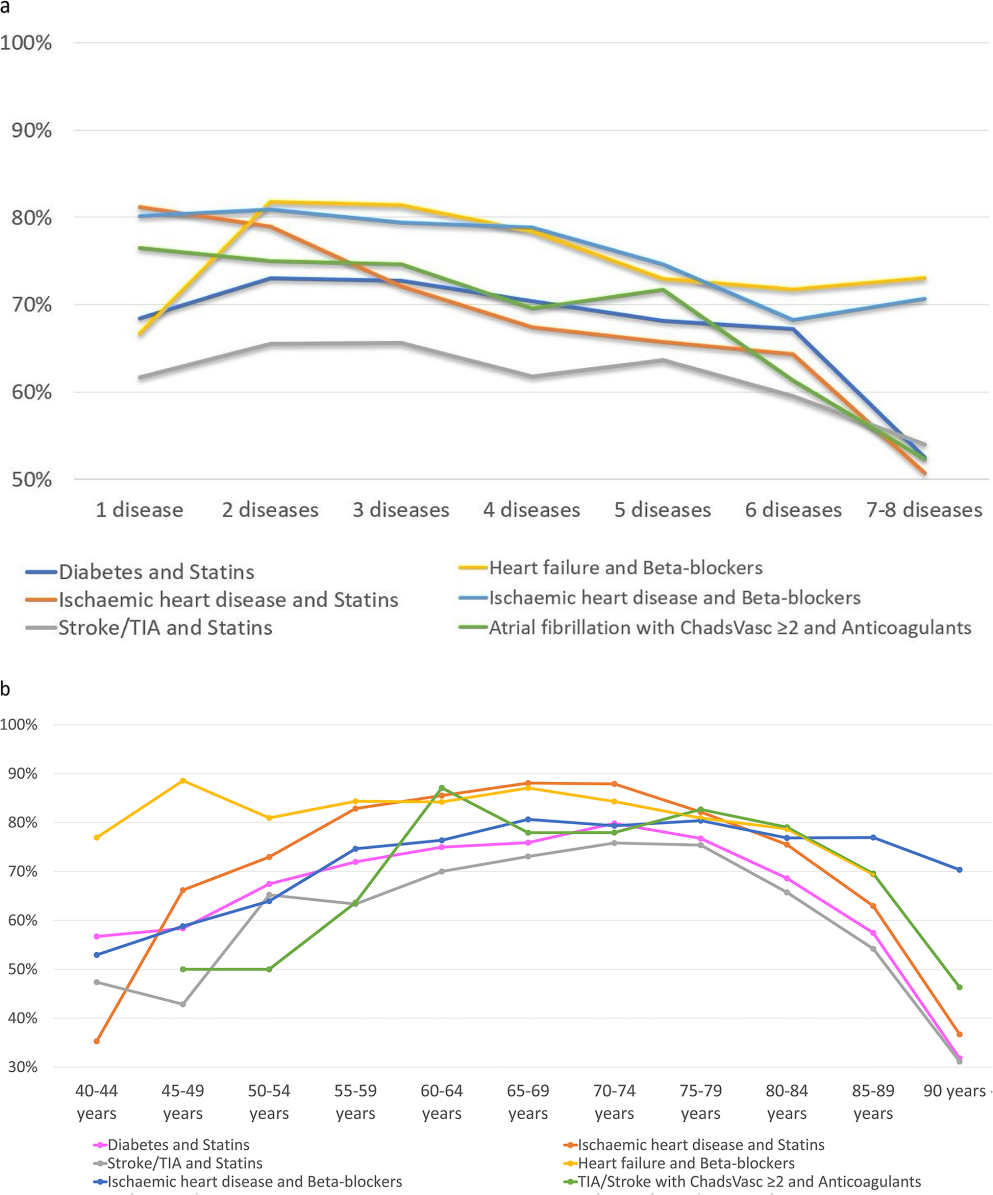
**a** Percentage of individuals with different number of diseases who received prescriptions of medication according to guidelines for diabetes, IHD, Stroke/TIA, Heart Failure, and Atrial Fibrillation + CHA2DS2-VASc > 2. **b** Percentage of individuals in different ages who received prescriptions of medication according to guidelines for diabetes, IHD, Stroke/TIA, Heart Failure, and Atrial Fibrillation + CHA2DS2-VASc > 2

 Patients with diabetes, ischaemic heart disease and stroke/TIA were considered as having an indication for statins. The more diseases these patients had, the fewer were prescribed statins (73% of patients with 1–2 diseases, 52% with 7–8 diseases). Similarly, for patients with atrial fibrillation and CHA_2_DS_2_VASc ≥ 2 (considered as having an indication for anticoagulants), the more diseases these patients had, the fewer were prescribed anticoagulants (75% of patients with 1–2 diseases, 52% with 7–8). For beta-blockers (patients with ischaemic heart disease and heart failure considered as having an indication), there was no such association (Fig. 3a).

The number of prescriptions also varied with age, with more prescribing of all three medications to patients around the age of 65–70 and less to younger and older (Fig. 3b).

### Continuity of care

The median number of GPs the patients met throughout the study period was 4 (mean 4.2) with a range between 1 (i.e., the same doctor at all visits) and 35 different GPs.

The continuity, measured as MMCI and COC, did not differ between genders, patients within different age groups, different diagnoses/diseases, different number of diagnoses/diseases, or different combinations of 2 or 3 diagnoses/diseases.

COC and MMCI were also approximately the same for patients with different number of visits. A separate analysis was done comparing patients with a very high number of visits with the rest: we compared 90-99th percentile and 99-100th percentile with the 0-90th percentile. This analysis was performed for patients with 1, 2, and 3 diagnoses/diseases. We found that, regardless of the number of diseases, COC decreased slightly for patients with many visits, while MMCI increased slightly, but the difference was very small (Table 4).

We did not find any difference in continuity (including all subgroups) between patients who were or were not prescribed medication according to guidelines.

**Table 4:** Continuity measured as MMCI and COC, respectively, for 1–3 diseases and for different numbers of visits (< 13 visits totally during the 4-year period, 13–24 visits and > 24 visits). Mean and standard deviations, p5-p95. and minimum and maximum values are shown

	N	mean	SD	p5	p50	p75	p95	min	max
1 disease									
COC	10,046	0.36	0.30	0	0.3	0.5	1	0	1
MMCI	10,046	0.56	0.28	0.03	0.56	0.76	1	0.01	1
2 diseases									
COC	9025	0.36	0.29	0	0.29	0.5	1	0	1
MMCI	9025	0.58	0.26	0.05	0.61	0.76	1	0.01	1
3 diseases									
COC	3999	0.36	0.27	0.04	0.29	0.5	1	0	1
MMCI	3999	0.61	0.23	0.18	0.62	0.78	1	0.01	1
1 disease, number of visits < 13									
COC	8900	0.37	0.31	0	0.3	0.5	1	0	1
MMCI	8900	0.55	0.28	0.03	0.52	0.76	1	0.01	1
1 disease, number of visits 13–24									
COC	1053	0.30	0.23	0.04	0.23	0.42	0.92	0	1
MMCI	1053	0.62	0.20	0.29	0.62	0.77	0.92	0.01	1
1 disease, number of visits > 24									
COC	93	0.29	0.22	0.05	0.24	0.36	0.74	0.04	0.83
MMCI	93	0.68	0.16	0.39	0.70	0.77	0.96	0.32	0.97

## Discussion

### Summary of the findings

The study shows that in the group of patients with common chronic diseases and regular visits to primary care, constituting around 7.5% of the population in the region, diabetes was the most frequent disease, and anxiety and depression together with diabetes and ischaemic heart disease were the most frequent combinations of two diseases. Multimorbidity was most prevalent in the oldest patient group. There was a slight positive correlation between number of diseases and visits, but with no linearity between increasing number of diseases and increasing number of visits. The number of visits varied slightly with different combinations of diseases, where patients with physical conditions combined with anxiety and/or depression made more visits. We found a negative association between adherence to guidelines concerning secondary prevention with statins and anticoagulants and number of diseases per patient. Patients with higher values of continuity were not more likely to be prescribed guideline-concordant medications.

### Methodical limitations

Chronic diseases are defined in different ways in different studies [2, 32, 33]. Sometimes only non-communicable diseases are included, but in other definitions also infectious diseases (e.g. HIV and hepatitis C) are included. In a systematic review on multimorbidity patterns, the most common included diseases were: COPD, diabetes, hypertension, malignancy, stroke, dementia, depression, joint disease, anxiety, and congestive heart failure [34]. Different definitions in different studies can be a problem and Trivedi emphasises in a Cochrane Review the importance of common definitions of comorbidity and multimorbidity [32].

However, our intent was to include common chronic diseases that are regularly monitored in Swedish primary care, because we wanted to study patients with these diseases with respect to continuity and adherence to guidelines on prescription of medication. Patients with the chronic diseases that are included in the study usually visit a GP regularly, at least by an annual check-up. Sometimes they also see primary care nurses regularly. However, most patients with these diseases do not see other specialists regularly, apart from GPs. Chronic diseases that are common reasons for visits to GPs but not monitored by annual check-ups in Swedish primary care (e.g., osteoarthritis) were not included. Some conditions, e.g., hypertension and hyperlipidaemia, can be considered as risk factors rather than diseases [35], and these conditions are often monitored by specialised practice nurses and the patients do not always see a GP every year. Also, diseases normally monitored by other specialists than GPs, such as rheumatoid arthritis and most malignancies, were not included in this study.

All GPs in the region use the same EMR system, and data from the system is generally accurate. However, the number of diagnoses in the study population may be affected by the ACG system used for reimbursement for primary care in Sweden. Studies have shown that ACG is sensitive to the accuracy of coding of diagnoses by physicians, and patients tend to get more diagnoses when the ACG system is used [36–38].

The frequent multimorbidity and the diseases found together in our study reflect the selection of patients in the study. We included only patients with chronic diseases and 3 or more visits during the study period to obtain a sample of patients with relatively high needs of care.

The analyses did not control for confounding factors as e.g. health literacy, education level, health behaviour or distance to the PHCC as these data were not available on the individual level.

At least three visits in the study period were considered as an indication of need of continuous care. For patients with chronic diseases, but fewer visits (i.e., less than once a year) continuity in primary care is less relevant to measure. These patients might have more severe disease and see other specialists more regularly, or their diseases are less severe, and they do not need regular care. However, there might be patients with the selected chronic diseases also among those who did not have at least three visits during the period and including them might have rose the numbers of patients with diseases and multimorbidity.

A review of indices for continuity of care reveals a significant variation in 32 identified indices based on different aspects such as duration, density, dispersion, sequence, and subjective estimates. Thus, there has been no consensus in the literature about what should comprise continuity of care indices, and no index is wholly inclusive of all facets of continuity [21]. It remains therefore difficult to grasp the essence of continuity of care in one “superior“ index. The various calculations of continuity of care have e.g. included patients’ experiences of continuity or basic structural information such as registration with a specific primary care doctor. This poses certain obstacles for data retrieval and limitations when comparing results. The availability of complete population data in EMRs, with full coverage of all primary care visits to specific doctors enables researchers to accomplish more accurate investigations.

### Prevalence of common chronic diseases and multimorbidity

The prevalence of multimorbidity in this study is much lower than the prevalence from other studies described in a systematic review by Fortin et al. due to the very limited number of common diseases that were included. The relatively low comorbidity between diabetes and depression, compared to other literature and the high comorbidity rate between depression and anxiety, may be explained by the fact that depression is less likely to be recognized in medical morbidities such as diabetes (although the actual prevalence of depression in patients with diabetes is significantly higher than that in the general population) [39]. Several authors have presented the epidemiology of multimorbidity including common combinations of diseases [15, 34, 40]. Our results show the pattern for patients with chronic diseases who are seen regularly by GPs and for whom GPs also have the main treatment responsibility. In this group of patients, multimorbidity is much more common than single diseases. Thus, primary care needs to focus on this group concerning quality improvement and adaptation of guidelines. The combination of diabetes and/or heart disease (heart failure and/or ischaemic heart disease) and depression is often addressed in programmes aimed to improve management for people with mixed mental and physical multimorbidity [33, 41, 42]. This group accounted for almost 20% of the study population.

### Primary care utilisation

Patients in our study made an average of 2.1 visits to a GP each year. This is internationally a low number, especially since our study population all had chronic diseases that normally need regular check-ups by a GP. However, it is not an exceptional figure in Sweden where the total population average is 1.5-2 visits to a GP per year (and around 1.5 additional visits to other specialists per year) [26]. There are several possible explanations for this, e.g., longer visits, active referral to other competences at the primary health care centre (PHCC) in a triage cooperation within the centre, resulting in more visits to practice nurses, physiotherapists, and other staff at the PHCC.

Persons with multimorbidity had higher consultation rates according to a study by Salisbury [43]. In our study the number of visits varied greatly. A few patients made an extensive amount of visits and the majority made a few. Patients with more diseases made more visits but not in proportion to the number of diseases. This could be understood as a higher degree of effectiveness in primary care as several problems could be dealt with in one visit compared to if the patients had to visit one specialist for each disease.

Patients with multimorbidity including anxiety and/or depression made more visits than patients with other comorbidities. This could be understood as these patient groups needed more frequently their GPs´ attention or advice. The effective implementation of collaborative care models for management of depression in primary care which have shown to be effective in improving mental health outcomes could reduce the number of GP consultations.

### Adherence to guideline-based pharmacotherapy

As indicators of adherence to guidelines, prescriptions of beta-blockers and/or statins were chosen for patients with diabetes, ischaemic heart disease, heart failure and TIA/stroke. Similarly, for patients with atrial fibrillation (and indication for treatment i.e. CHA_2_DS_2_VASc ≥ 2), prescription of anticoagulants was analysed. Almost one-third of the patients with diabetes, ischaemic heart disease, heart failure, and TIA/stroke were not prescribed medication according to guidelines. However, this might be appropriate in the individual cases due to contraindications or other limitations. The aim of Swedish National guidelines for secondary prevention is to attain treatment rates between 65-90% of the patients depending on disease, age, and type of medication [10, 11].

The finding that patients with many diseases received fewer prescriptions of statins and anticoagulants might indicate the GPs´ consideration of contraindications, limitations, and the risks of polypharmacy in individual cases. The “peak” in prescribing to patients around the age of 70 might reflect less severe disease in younger patients and avoiding over-medication in older patients. However, it remains unclear in our study if medications were appropriate in these different groups of patients.

Prescribing according to guidelines is commonly used as a quality indicator [26]. However, as several studies indicate that following single disease guidelines in patients with multimorbidity often causes problems such as polypharmacy and adverse drug events, high costs, and very complex self-care regimes [13, 15, 16], this type of indicators may not be useful in a population with high prevalence of multimorbidity. Our results indicate that the prevalence of multimorbidity is high and thus prescribing according to guidelines is not an appropriate way to measure quality in primary care. Care for these patients is complex and needs to be individualised [44–46]. For example, in a Swedish study half the patients who had indication for anticoagulant treatment according to CHA_2_DS_2_VASc score had complicating co-morbidities that made treatment questionable [47]. An increasing and important task for primary care is, by using patient-centred and holistic care, balancing these tasks as described in the current NICE multimorbidity guidance [1]. Our results with lower prescribing according to guidelines for old and multimorbid patients indicate that this might be the case.

### Continuity of care

In the above mentioned Salisbury et al. study, persons with multimorbidity had less continuity of care compared with people without multimorbidity (measured as usual provider continuity index and the continuity of care index) [43]. A possible explanation could be that patients with multimorbidity have more GP contacts, which might make continuity more difficult. In our study, where the entire study population had between one and eight chronic diseases each, the two continuity indices did not differ among patients with different numbers of diseases or different subgroup characteristics (sex, age, type of diseases, combinations of diseases, and number of visits). Moreover, continuity was not associated to adherence to guidelines for secondary prevention. These findings are in contrast to earlier research findings that show an association between continuity and healthcare utilisation and the quality of care [48]. However, quality of care remains difficult to evaluate through these measures, as adherence to guidelines in secondary prevention does not seem to reflect quality of care in primary care for patients with multimorbidity. Patients who were well-known by their GP might have received a more person-centred, individualised care with appropriate medication, including the omission of medicines due to age, multimorbidity, risks and/or other individual preferences and features, than treatment according to the many silo guidelines.

As continuity is a complex phenomenon, the two chosen continuity indices might not reflect “good continuity” in primary care. Patients may experience continuity even if they do not see their GP every visit, e.g. if they think there is good communication between their GP and the temporary one, or if their GP keeps in contact by phone. Perhaps seeing the same nurse or someone else in the care team (collaborative care) makes the patients experience continuity. It is also possible that the other components of continuity, i.e. informational continuity and management continuity, are as important as relationship continuity [25], and these dimensions were not included in the indices. Research with extensive databases needs to be undertaken for further understanding.

### Conclusions concerning evaluation and improvement of quality of care

With almost all patient records now digitalised, there is a risk of choosing too simple, mechanised, and ostensibly fair but inaccurate ways to measure complex issues. The risk of the silo perspective, where each diagnosis is assessed and evaluated separately is not applicable in the group of patients with multimorbidity, a high prevalence group with the high care need in primary care. New, appropriate, and more comprehensive ways of assessments of quality of care and improvements in the management of patients with chronic diseases and multimorbidity, where a more person-centred perspective also is included, have to be developed for primary care and healthcare as a whole.

### Supplementary Information

**Additional file 1.**

## Data Availability

The datasets used and/or analysed during the current study are available from the corresponding author on reasonable request.
