# Supplementary material for: Primary care utilisation, adherence to guideline-based pharmacotherapy and continuity of care in primary care patients with chronic diseases and multimorbidity – a cross-sectional study
Source: BMC Prim Care. 2023 Nov 13;24:237. doi: 10.1186/s12875-023-02191-6 (PMC10644564; doi:10.1186/s12875-023-02191-6)
Supplement: Supplementary file 1 — Additional file 1. [file 12875_2023_2191_MOESM1_ESM.docx]

# Additional file 1

**Appendix 1**

ICD10 Diagnosis codes:

Anxiety: F40.0-9, F41.0-9, F42.0-9, F43.0, F43.8, F43.9, F43.0-9, F48.8

Dementia: F00.1-F03.9, F10.7, F10.7A, G30.0-G30.9

Depression: F32.0-F32.9, F33.0-F33.9, F34.1-F34.8, F34.9, F38.0 - F38.8, F39.9, F43.1, F43.8, Z73.0

Diabetes: E10.0-E11.9, E13.0- E14.9

Atrial fibrillation: I48.0-I48.9

Heart failure: I11.0, I13.0, I13.2, I42.0, I50.0-I50.9

Ischemic heart disease: I20.0- I23.2, I23.8- I25.9

COPD: J44.0-J44.9

Stroke/TIA, Ischemic: G45.0-G45.3, G45.8-G45.9, I63.0-I63.9, I64.9, I67.8-I67.9, I69.3-I69.8, Z86.6A-Z86.6B, Z86.7C and Haemorrhagic: I61.0-I61.9, I69.1

Vascular disease : I70,0-I70,9, I73.9, I73.9B, I74.0-I74.9 (For calculations of CHADSVASC also codes for Ischemic heart disease as defined above are included, see appendix 3)

**Appendix 2**

ATC-codes:

Statins: C10AA01, C10AA03, C10AA04, C10AA05, C10AA07

Beta-blockers: C07AA03, C07AA05, C07AA07, C07AB02, C07AB03, C07AB07, C07AB08, C07AB12, C07AG01, C07AG02

Anticoagulants: B01AA03, B01AE07, B01AF01, B01AF02

**Appendix 3**

ChadsVASC

Patients with atrial fibrillation (ICD10 I48.0-I48.9) and CHADSVASc ≥ 2 were considered as having an indication for anticoagulants. CHADSVASc score was calculated according to table below.

| **Criteria** | **Score** | **ICD-10 codes** |
| --- | --- | --- |
| Heart Failure | 1 | I11.0, I13.0, I13.2, I42.0, I50.0-I50.9 |
| Hypertension | 1 | I10.9, I11.9, I12.0-I12.9, I13.9, I15.0-I15.9 |
| Age ≥75 years | 2 |  |
| Diabetes Mellitus | 1 | E10.0-E11.9, E13.0- E14.9 |
| Stroke/TIA/ Thromboembolism | 2 | G45.0-G45.3, G45.8-G45.9, I63.0-I63.9, I64.9, I67.8-I67.9, I69.3-I69.8, Z86.6A-Z86.6B, Z86.7C, I74.0-I74.9 |
| Vascular Disease | 1 | I70.0-I70.9, I73.9, I73.9B, I20.0- I23.2, I23.8- I25.9 |
| Age 65-74 years | 1 |  |
| Female | 1 |  |
